# OLDER PARENTS’ PATTERNS OF AGREEMENT AND INFORMATION SHARING WITH ADULT CHILDREN AND THEIR EMOTIONAL OUTCOMES

**DOI:** 10.1093/geroni/igad104.3067

**Published:** 2023-12-21

**Authors:** Noriko Toyokawa

**Affiliations:** Southern Oregon University, Ashland, Oregon, United States

## Abstract

The current study examined the effect of older parents’ pattern of agreement/disagreement with their adult children’s perceptions of their functional ability and their information disclosure/non-disclosure to their adult children on their ambivalent, positive, and negative emotions. Older parents, N=263, Mage=75.06, SD=5.49, participated in an online survey. Participants’ agreement with adult children’s ideas and caregiving practice to support 10 life issues were assessed by five categories: 1=agree, 2=disagree, 3=it depends, 4=it is not my issue, and recorded into 0=agree, 1=fully/partially disagree/not my issue. Participants’ information sharing on the same items was assessed by 1=openly share, 2=leave out critical points, 3=avoid talking about, 4=lie or making a story, 5=It is not my issue, and recoded to share 1=openly and 0=not openly share. The median dichotomized the mean scores of 10 items to create four patterns: disagree-do not disclose, disagree-disclose, agree-do not disclose, and agree-disclose. Positive, negative, and ambivalent emotions toward adult children have been assessed by the scales and formula used by Lendon et al. (2013). A series of one-way ANOVA, F(3, 260)=4.41, p=.005, η=.05, revealed that participants in agree-do not disclose the pattern, M=3.15, SD=.90, reported the highest level of ambivalence. A post hoc test showed it was significantly higher than that of participants in disagree-did not share, M=2.16, SD=1.10. Disagree-do not disclose group reported the lowest score in positive and negative emotions among groups. Frequency of contact predicted disclosure in auxiliary analysis. The need for within and between differences in older parents’ agreement and disclosure patterns was discussed.

